# The relationship between eccentric hamstring strength and eccentric absorption during the landing phase of vertical jumps in soccer players

**DOI:** 10.3389/fphys.2026.1886499

**Published:** 2026-07-08

**Authors:** Sibel Yildirim, Fırat Akyüz, Nihat Özşengezer, Atilla Orkun Dilber, Mehmet Ali Ekin, Kıvanç Buru, Murat Akyüz

**Affiliations:** 1 Faculty of Sports Sciences, Hitit University, Çorum, Türkiye; 2 Vocational School, Ağrı İbrahim Çeçen University, Ağrı, Türkiye; 3 Faculty of Sports Sciences, Manisa Celal Bayar University, Manisa, Türkiye; 4 Sarıkamış Faculty of Sports Sciences, Kafkas University, Kars, Türkiye

**Keywords:** eccentric strength, energy absorption, hamstrings, landing mechanics, soccer

## Abstract

**Objective:**

This study aimed to examine the relationship between eccentric hamstring strength and biomechanical energy absorption parameters during the landing phase of a vertical jump, and the impact of a pre-season preparation period on these variables in professional soccer players.

**Methods:**

Thirty-one professional male soccer players (n=31, age: 24.81±5.23 years) participated in the study. Eccentric hamstring strength was measured using the NordBord system, and countermovement jump (CMJ) landing kinetics were assessed via ForceDecks dual force plates before and after the pre-season camp. The landing phase was analyzed through Peak Landing Force, Landing Impulse, and Landing Rate of Force Development (RFD).

**Results:**

After the preparation period, a significant decrease in Peak Landing Force (5429.89±1865.7N vs. 4939.13±1449.8N; p=0.012) and a significant increase in Landing Impulse (98.2±18.19 Ns vs. 106.54±24.67 Ns; p=0.029) were observed. Furthermore, a statistically significant reduction of 11.6% was found in Landing RFD (p=0.047). A statistically significant increase of 7.4% was observed in normalized Nordic hamstring strength (p=0.021).

**Conclusion:**

The pre-season training period enabled soccer players to develop a “softer” and more controlled landing strategy by enhancing the eccentric braking capacity of the hamstrings. The reduction in impact forces and the increase in impulse indicate more effective absorption of loads on the knee joint. Integrating eccentric hamstring exercises and landing mechanics drills into training programs is recommended to reduce injury risk.

## Introduction

Modern soccer is a high-intensity, dynamic sport that requires players to perform countless sudden accelerations, sprints at maximum speed, changes of direction, and explosive vertical jumps throughout a match (Bangsbo et al., 2006; Mjølsnes et al., 2004). Within these dynamics, the vertical jump is a fundamental element that determines the outcome of the game, particularly in both offensive and defensive actions such as aerial duels (Chrisman et al., 2012; Bisciotti et al., 2019). While there is significant focus in the literature on the “ascent” phase of performance, the landing phase following the jump is of critical importance—at least as much as jump height—from the perspectives of athlete health and the sustainability of performance (Pollard, Sigward, & Powers, 2009; Jakobsen et al., 2012). Increases in vertical jump performance lead to higher ground reaction forces and impact loads at the moment of landing, as the center of mass falls from a greater height. This situation makes the importance of the hamstrings’ eccentric control mechanisms even more critical in meeting the demands of increased performance. Landing mechanics is a brief, energy-absorbing, and predominantly eccentric process in which the kinetic energy gained by the body’s center of mass is dissipated by the lower extremity muscle-tendon units (Dewig et al., 2020; Vogt and Hoppeler, 2014; Norcross et al., 2013). During this millisecond timeframe, ground reaction forces (GRF)—which can reach several times the body’s weight—must be safely managed (VALD Performance, 2025). When energy absorption is insufficient, mechanical loads are transferred uncontrollably to the passive structures of the knee joint, particularly the anterior cruciate ligament (ACL) (Gaugg et al., 2025; Zanguie et al., 2024; Hewett et al., 2005). At this point, the eccentric strength capacity of the hamstring muscle group—which stabilizes both the hip and knee joints through its bi-articular structure—emerges as a “biomechanical braking system” (Behm et al., 2015; Umberger, 1998). Current evidence confirms that athletes with high eccentric hamstring strength can decelerate their center of mass more controllably and minimize the instantaneous loads on the knee joint (Dewig et al., 2020; Severo-Silveira et al., 2018). Conversely, insufficient eccentric strength or lower-body asymmetry leads to a “stiff landing” strategy in the lower extremities, significantly increasing the risk of injury (Benson et al., 2024; Blackburn et al., 2003). Furthermore, since athletes’ absolute strength capacities can be influenced by changes in body mass, normalizing strength data by body weight (N/kg) provides a more objective reflection of actual muscle adaptation and functional performance (Sinulingga and Zemková, 2025; Jaric, 2002). Most injuries that occur during the landing phase of a jump take place within the first 50–100 milliseconds, when ground reaction forces peak (Studnicki et al., 2025). Since this limited time window is shorter than the time required for the muscle to reach maximum force production, the rate at which force is generated—that is, Rate of Force Development (RFD)—rather than “absolute force,” is the key to stabilization (Laffaye and Wagner, 2013; Cowell et al., 2012). The hamstrings’ eccentric RFD capacity in this process creates a dynamic shield against sudden loads (Severo-Silveira et al., 2018). However, the extent to which force data obtained from isolated tests, such as the Nordic Hamstring Exercise (NHE), can be transferred to this dynamic energy absorption task still requires comprehensive examination in the literature (Aka et al., 2026; Alt et al., 2022).

Reviews of strategies for preventing hamstring injuries in soccer emphasize that eccentric training not only increases muscle strength but also modifies muscle architecture to create a protective structure (Bisciotti et al., 2019; Attar et al., 2016). It has been reported that athletic performance programs implemented during training camp significantly improve soccer players’ strength, jumping, and balance parameters (Hosseini et al., 2025). Although it is known that NHE protocols positively influence landing kinetics (Severo-Silveira et al., 2018; Krommes et al., 2017), the relationship between this process and energy absorption strategies (impulse and RFD management) in professional soccer players still requires in-depth investigation. In this context, the aim of this study is to evaluate the relationship between eccentric hamstring strength and energy absorption parameters during the landing phase of a vertical jump in elite soccer players.

## Methods

### Participants

The sample for this study consisted of 31 male athletes (n=31) actively playing for a professional soccer club. The study group was selected from athletes who fully participated in the preseason training camp and took part in all team training sessions before and after the camp. The participants’ demographic characteristics—age (24.81 ± 5.23 years), height (181.2 ± 6.4 cm), and body weight (pre-test: 75.42 ± 5.49 kg; post-test: 73.42 ± 5.92 kg)—were recorded. Inclusion criteria required that participants had not sustained any musculoskeletal injury affecting the lower extremities (hip, knee, or thigh) within the past 6 months and were in full health capable of performing the test procedures at maximum effort. All participants were provided with detailed information about the purpose of the tests, and informed consent was obtained.

### Data collection and procedures

All measurements were taken on the natural grass field where the athletes conduct their routine training, under standard weather conditions and at similar times of day (**Table 1**).

**Table 1:** Training distribution for the 6-week preparation camp.

Week	Weekly session	Number of sets and repetitions	Weekly total reps
1st. Week	1	2 sets x 5–7 reps	10-14
2.nd Week	1	2 sets x 6–8 reps	12-16
3rd. Week	2	3 sets x 8–10 reps	48-60
4th. Week	3	3 sets x 8 reps	72
5th. Week	3	3 sets x 10 reps	90
6th. Week	3	3 sets x 12-10–8 reps	90

The Nordic Hamstring (NHE) protocol used in this study was based on the principles of progressive loading described by Mjølsnes et al. (2004). During the training camp, the athletes followed a program in which the number of weekly sessions and the number of sets and repetitions were gradually increased. During the first two weeks, neuromuscular adaptation was achieved with 1 session per week (2 sets x 5–7 repetitions); in weeks 3 and 4, the number of weekly sessions was increased to 2 (2–3 sets x 8–10 repetitions). Over the past two weeks, the training volume was set at 3 sessions per week (3 sets of 12–10–8 repetitions). This was structured according to a traditional pyramid loading method; in this method, rather than intentionally fatiguing the athletes’ muscles, the target number of repetitions was systematically reduced across consecutive sets to support the maintenance of eccentric speed and movement control. During the exercise, athletes were instructed to keep their hips in a slight flexion, use their hamstring muscles to resist the forward fall for as long as possible, and minimize concentric loading by using their hands at the moment of contact with the ground. Athletes who missed a training session were required to complete the protocol individually on the same day, ensuring a high level of compliance.

#### Eccentric hamstring strength test

Athletes’ eccentric knee flexor (hamstring) muscle strength was assessed using the NordBord (VALD Performance, Queensland, Australia) hamstring testing system (Rhodes et al., 2025). During the measurement, athletes were positioned on the device’s padded surface in a kneeling position, with their ankles securely fastened to hooks equipped with integrated load cells. The athletes were instructed to maintain their trunk and hips in a neutral position, lean forward at the slowest possible speed, and exert maximum resistance against gravitational acceleration using their hamstring muscles (Alt et al., 2022; Šarabon et al., 2019). At the point where the muscle’s eccentric force was released, the athletes used their hands to soften the contact with the ground and returned to the starting position. The device simultaneously recorded the peak eccentric force (Peak Force) produced by both legs in Newtons (N).

#### Vertical jump and landing test

The biomechanical parameters of soccer players during the vertical jump and landing phases were obtained using the ForceDecks device (VALD Performance, Queensland, Australia), which features dual-platform technology, via the Countermovement Jump (CMJ) test (Jakobsen et al., 2012). The athletes were instructed to step onto the platform with their feet shoulder-width apart and hands on their hips, perform a rapid squat, immediately follow it with a maximum-effort vertical jump, and then land back on the platform in a controlled manner (Jakobsen et al., 2012; Krommes et al., 2017; Ferguson et al., 2023). Kinetic data for the landing phase—specifically Peak Landing Force, Landing Impulse, and Landing Rate of Force Development (Landing RFD)—were analyzed using the device’s reference values as the baseline.

### Statistical analysis

Eccentric hamstring force and peak descent force data were normalized by dividing them by body weight (Newton/kg) to account for potential changes in each athlete’s body mass (BM). These relative force values were used to assess the actual effect of the training period on muscle adaptation (Sinulingga and Zemková, 2025). The analysis of the obtained data was performed using the SPSS 26.0 (IBM, USA) software package. The Shapiro-Wilk test was used to determine whether the data followed a normal distribution. Differences between pre- and post-tests were analyzed using the Paired Samples t-test for normally distributed data and the Wilcoxon Signed-Rank test for non-normally distributed data. The Spearman correlation coefficient (r) was used to determine the relationships between variables. A significance level of p<0.05 was accepted.

## Results

Descriptive statistics and comparative analyses were conducted for the professional soccer players (n=31) who participated in the study, based on data collected before (pre-test) and after (post-test) the preseason training camp.

### Demographic and descriptive characteristics of the participants

Upon evaluating the demographic data and physical characteristics of the professional soccer players included in the study, the participants’ mean age was found to be 24.81 ± 5.23 years, and their mean height was 181.25 ± 6.41 cm. The average body weight of the athletes at the start of the preseason training camp was 75.43 ± 5.49 kg, while this value decreased to 73.42 ± 5.93 kg by the end of the camp. The athletes’ average Body Mass Index (BMI) was calculated as 22.95 ± 1.14 kg/m², and it was determined that all athletes fell within the ideal physical fitness standards required for professional soccer (**Table 2**).

**Table 2:** Demographic and physical characteristics of participants (n=31).

Parameters	Min	Max	Average (Xˉ)	Standart Deviation (SD)
Age (years)	18.00	36.00	24.81	5.23
Height (cm)	169.00	192.00	181.25	6.41
Body Weight - Pre-Test (kg)	64.95	85.85	75.43	5.49
Body Weight - Post-Test (kg)	60.29	84.60	73.42	5.93
Body Mass Index (kg/m²)	20.12	24.63	22.95	1.14

### Comparison of hamstring strength and landing biomechanics parameters

**Figure 1:**
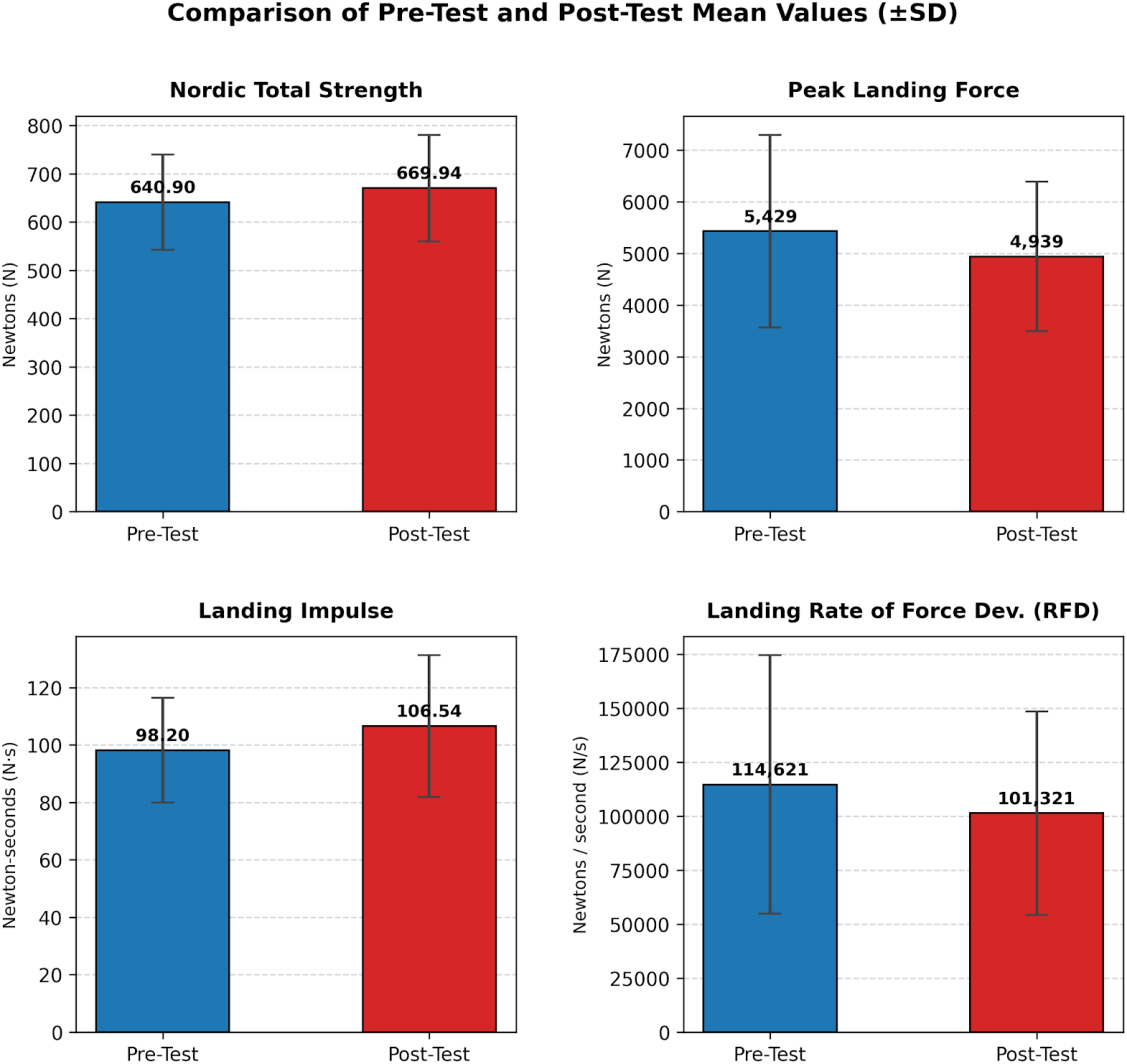
Relative percentage changes from pre-test to post-test across performance and biomechanical metrics. Positive values (green bars) represent postseason improvements/increases, whereas negative values (red bars) denote reductions. Significant post-training adaptations include a 4.5% increase in Nordic Total Strength (N), an 8.5% increase in Landing Impulse (Ns), a 9.0% reduction in Peak Landing Force (N), and an 11.6% reduction in Landing RFD (N/s). All highlighted changes represent statistically significant pre-to-post differences (p<0.05).

Changes in athletes’ eccentric hamstring strength and kinetic data from the landing phase of the CMJ were evaluated (**Table 3**). The analysis revealed a significant decrease in Peak Landing Force (p=0.012) and a significant increase in Landing Impulse (p=0.029) following the preseason training camp. Additionally, a statistically significant decrease was observed in Landing RFD values (p=0.047). The 4.5% increase in total Nordic hamstring strength was found to be near the threshold of statistical significance (p=0.060) (**Figure 1**).

**Table 3:** Comparison of pre-test and post-test absolute kinetic parameters (n=31).

Variables	Pre-Test (Xˉ ± SS)	Post-Test (Xˉ ± SS)	Changes (%)	t/z	P Value (Holm-Adj.)	95% CI (Lower, Upper)	Cohen’s d
Nordic Total Force (N)	640.90** ± **98.62	669.94 \ ± 110.55	+4.5%	-1.95	0.060	(-59.43, 1.35)	0.28 (Small)
Peak Landing Force (N)	5429.89 ± 1865.7	4939.13 ± 1449.8	-9.0%	-2.66	0.012*	(111.45, 870.07)	0.29 (Small)
Landing Impulse (N/s)	98.20 ± 18.19	106.54 ± 24.67	+8.5%	-2.29	0.029*	(-15.82, -0.86)	0.38 (Small)
Landing RFD (N/s)	114.621 ± 59.788	101.321 ± 47.066	-11.6%	-2.07	0.047*	(195.40, 26.404)	0.25 (Small)

*indicates statistical significance. *p<0.05 indicates statistically significant differences after Holm-Bonferroni correction. CI, Confidence Interval; RFD, Rate of Force Development.

When all the data obtained are evaluated as a whole, it is observed that the implemented program increased the athletes’ maximum strength capacity while simultaneously minimizing impact loads (peak force and RFD) during the landing phase.

#### Normalized nordic hamstring strength (N/kg)

**Figure 2:**
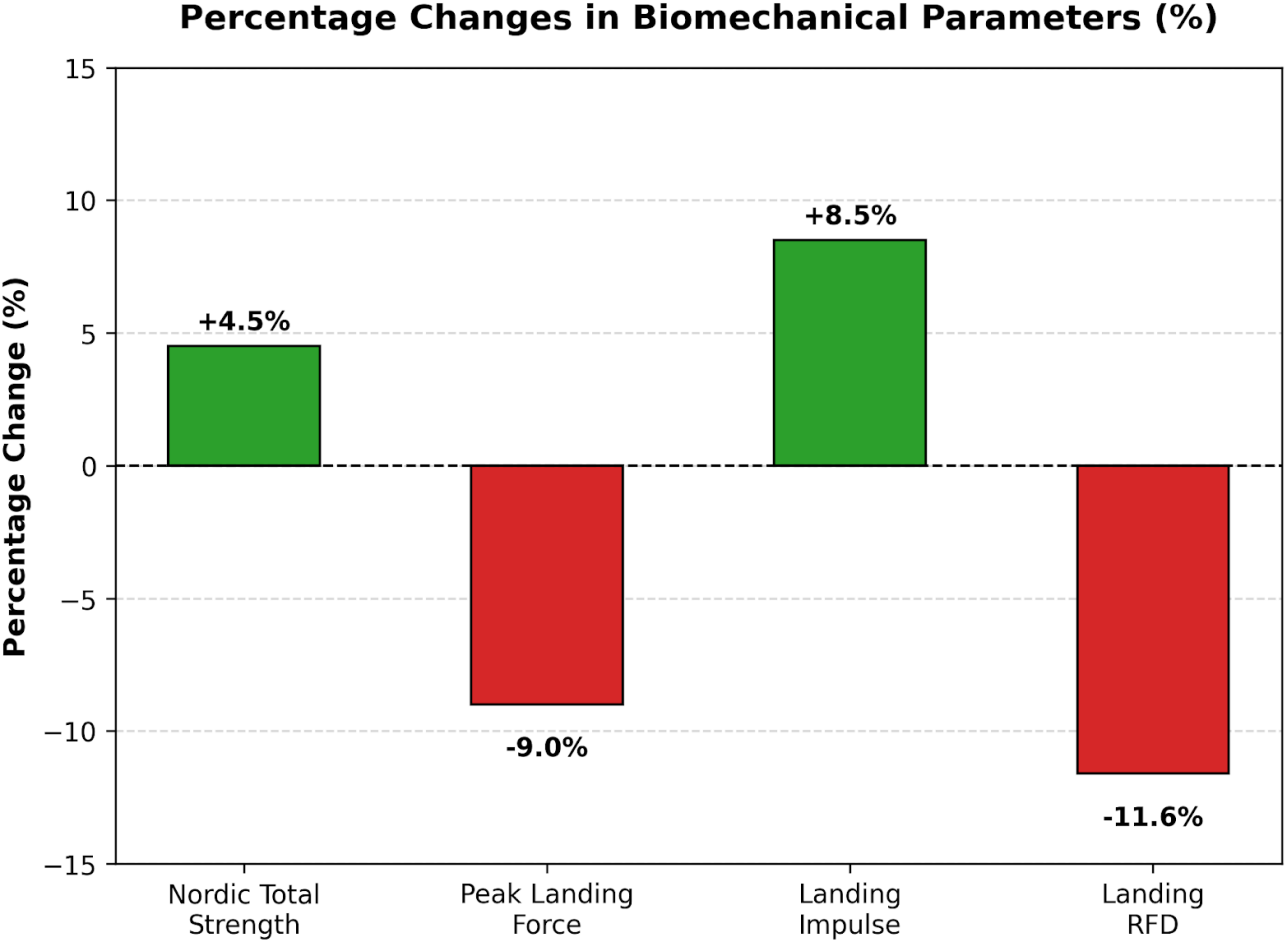
Changes in Nordic hamstring strength and countermovement jump landing biomechanics from pre-test to post-test. Bars represent the mean values for the pre-season preparation period (blue: pre-test, red: post-test), with error bars indicating ±1 Standard Deviation. Metrics evaluated include Nordic Total Strength (N), Peak Landing Force (N), Landing Impulse (Ns), and Landing Rate of Force Development (RFD, N/s). Significant improvements are observed post-intervention, reflecting an enhanced eccentric braking capacity and a more controlled landing strategy (p<0.05).

Athletes’ eccentric hamstring strength normalized to body weight was 8.49 ± 1.31 N/kg at the pre-test and was measured as 9.12 ± 1.51 N/kg at the end of the 6-week training camp (**Table 4**). The 7.4% increase in normalized Nordic strength relative to body weight (8.49 vs. 9.12 N/kg) was found to be statistically significant (p=0.021). Normalized Peak Landing Force (N/kg): The peak force per body weight at the moment of landing decreased from 71.98 ± 24.7 N/kg in the pre-test to 67.27 ± 19.7 N/kg in the post-test. This 6.5% decrease at the moment of landing was found to be statistically highly significant (p=0.001). Normalized Landing Impulse (Ns/kg): The normalized total momentum change during the landing phase increased from 1.30 ± 0.24 Ns/kg in the pre-test to 1.45 ± 0.33 Ns/kg in the post-test. This 11.5% increase was statistically significant (p=0.015). Landing Rate of Force Development (Landing RFD - N/s): The rate of force increase per unit time at the moment of landing was 114.493 ± 43.516 N/s in the pre-test, while it decreased to 101.236 ± 47.066 N/s in the post-test. This 11.5% decrease represents a statistically significant improvement (p=0.048) (**Figure 2**).

**Table 4:** Pre- and post-test comparison of parameters normalized by body weight (n=31).

Variables	Pre-Test (X ± SS)	Post-Test (X ± SS)	Changes (%)	t/z	p Value (Holm-Adj.)	95% CI (Lower, Upper)	Cohen’s d
Normalized Nordic Force (N/kg)	8.49 ± 1.31	9.12 ± 1.51	+7.4%	**-2.44**	0.021*	**(-1.16, -0.10)**	**0.44 (Medium)**
Normalized Peak Landing Force (N/kg)	71.98 ± 24.7	67.27 ± 19.7	-6.5%	**-3.62**	0.001*	**(2.05, 7.37)**	**0.21 (Small)**
Normalized Landing Impulse (Ns/kg)	1.30 ± 0.24	1.45 ± 0.33	+11.5%	**-2.58**	0.015*	**(-0.27, -0.03)**	**0.52 (Medium)**
Landing RFD (N/s)	114.493 ± 43.516	101.236 ± 47.066	-11.5%	**-2.06**	0.048*	**(136.21, 26.377)**	**0.29 (Small)**

*indicates statistical significance. *p<0.05, p<0.001 indicate statistically significant differences after Holm-Bonferroni correction. Landing RFD presents absolute temporal force distribution as tracked on the dual-plate baseline system.

### Relationship between eccentric hamstring strength and landing parameters

**Figure 3:**
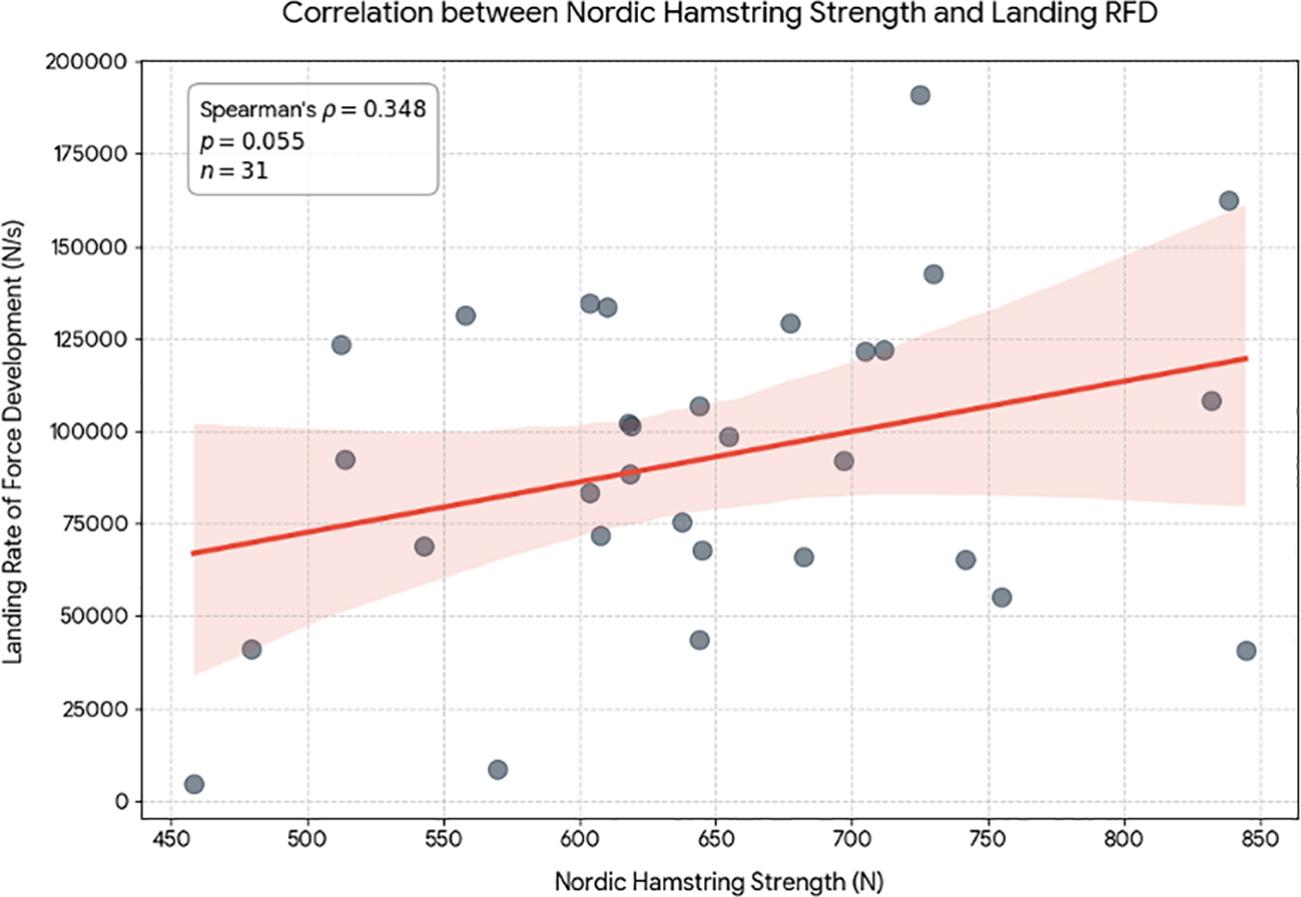
Scatter plot representing the correlation between eccentric Nordic hamstring strength and countermovement jump landing RFD. Individual data points represent professional soccer players (n=31). The trend line and its 95% confidence interval band indicate a positive correlation trend between the two variables, though the association narrowly missed the threshold for statistical significance (Spearman’s r=0.348, p=0.055).

**Figure 4:**
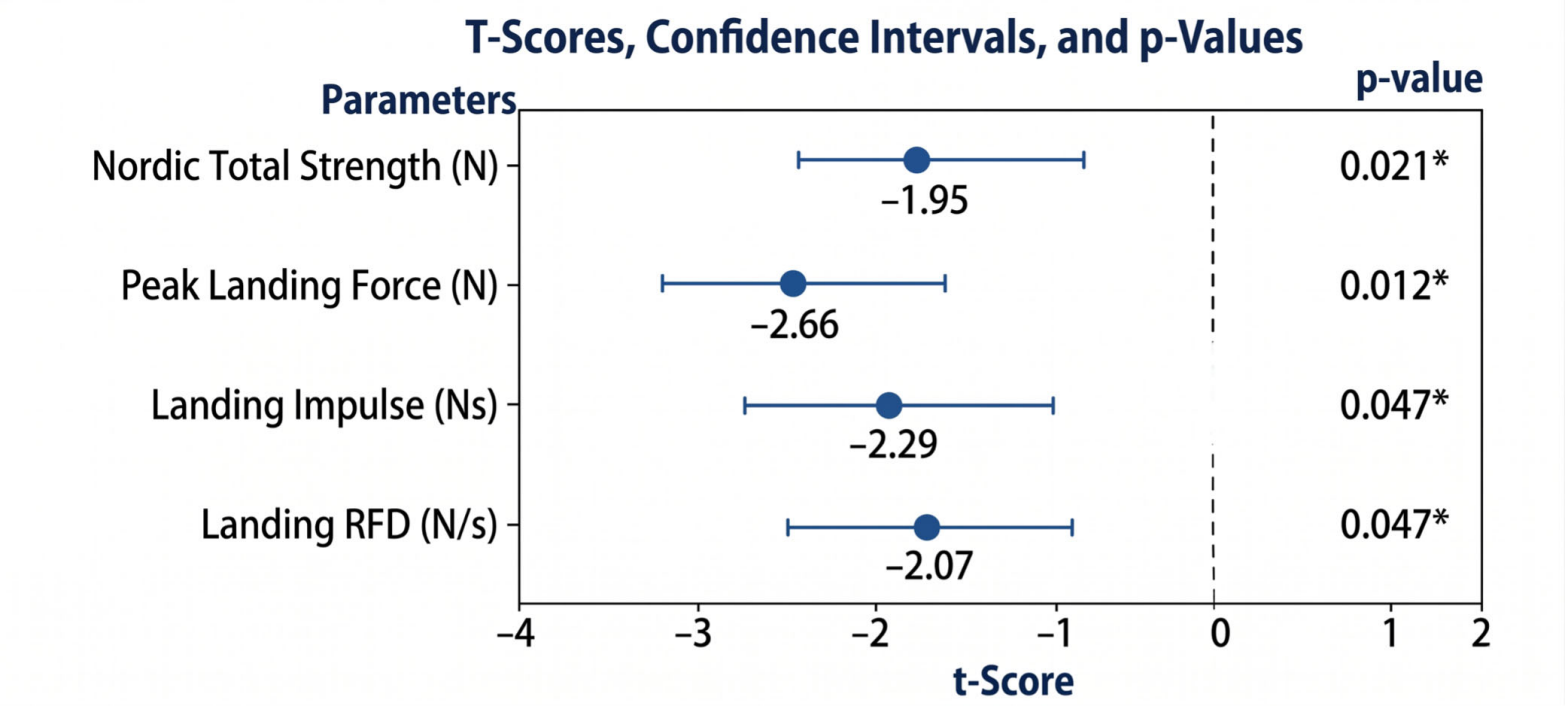
T-scores, confidence intervals, and corresponding p-values for the evaluated metrics. The plot illustrates the paired-samples t-test results comparing pre-test and post-test measurements. Central markers represent the calculated t-scores, with horizontal error bars indicating the 95% con!dence intervals of the difference. The dashed vertical line at 0 represents the null hypothesis line. All parameters demonstrated statistically significant changes following the pre-season preparation period, as indicated by asterisks. Exact p-values are provided on the right axis (*p<0.05).

A Spearman correlation analysis was conducted to determine the relationship between eccentric hamstring strength and landing phase parameters (**Table 5**, **Figure 3**). According to the analysis results, no strong direct linear correlation was found between hamstring force and landing parameters (p>0.05). However, a positive trend was observed between Nordic force and Landing RFD in the post-test data (r=0.348, p=0.055) (**Figure 4**).

**Table 5:** Correlation analysis between nordic hamstring strength and landing parameters (Spearman’s Rho).

Variables	Nordic force (Pre)	Nordic force (Post)
Peak Landing Force	r= 0.156, p = 0.39	r= 0.212, p = 0.25
Landing Impulse	r= 0.109, p = 0.55	r= 0.205, p = 0.26
Landing RFD	r= 0.217, p = 0.23	r= 0.348, p = 0.055

A moderate, positive, and statistically significant correlation was found between the height of the counter-movement jump (CMJ) and peak landing force (r = 0.524, p = 0.003). A positive and statistically significant correlation was also found between jump height and landing rate of force development (Landing RFD) (r=0.412, p=0.021). In contrast, no statistically significant relationship was found between jump height and the landing impulse (Landing Impulse) parameter (r=0.185, p=0.318).

## Discussion

The main findings of this study indicate that a preseason training camp for professional soccer players significantly improved not only isolated muscle strength but also dynamic landing strategies aimed at protecting the knee joint. The significant decrease in Peak Landing Force (p=0.012) and the increase in Landing Impulse (p=0.029) observed in athletes following the training camp demonstrate that the training period equipped the athletes with more effective deceleration and energy absorption capabilities. These changes serve as concrete indicators of the “soft landing” adaptation, which is defined in the literature as a critical safety parameter for elite athletes (Dewig et al., 2020; Severo-Silveira et al., 2018).

Pre-season training periods are critical phases during which soccer players maximize their physical capacity and integrate technical skills with neuromuscular coordination. The literature reports that athletic performance training implemented during training camps for professional soccer players yields multiple improvements in vertical jump, balance, and strength parameters. In our study, the observed trend of a 4.5% increase in total Nordic hamstring strength (p=0.060) is consistent with the general trend of strength development resulting from the training camp (Augustsson & Andersson, 2023; Hosseini et al., 2025; Lehnert et al., 2017). However, the primary improvement in our data appears to lie not in the muscle’s absolute strength capacity, but rather in the neuromuscular control of this strength during the “landing” phase—a dynamic task. However, when absolute strength data were normalized by body weight (N/kg), a statistically significant increase in body weight-normalized Nordic hamstring strength was observed (p=0.021). This disparity reveals that the observed ‘strength gain’ is not merely an expansion of absolute structural cross-sectional muscle force production but is largely a function of improved relative force production driven by significant body weight loss (75.43 kg to 73.42 kg) coupled with neural adaptations. This combination of systemic weight reduction and enhanced neuromuscular efficiency facilitated more effective damping of lower-extremity joint loads during sudden decelerations (Sinulingga and Zemková, 2025). In contrast, the significant decrease in normalized peak landing force (p=0.001) suggests that the reduction in athletes’ body weight, coupled with the concurrent increase in normalized force, facilitated more effective damping of joint loads and improved impact absorption quality.

Comprehensive studies on the prevention of hamstring injuries in soccer emphasize that eccentric strength capacity is not merely a performance metric but also a protective factor that modifies muscle-tendon architecture (Bisciotti et al., 2019; Attar et al., 2016). Bisciotti et al. (2019) argue that eccentric-focused training modalities minimize the risk of non-contact lower-limb injuries by significantly enhancing dynamic stability and neuromuscular feedback loops in the knee joint. The significant 11.6% decrease in Landing RFD (Rate of Force Development) observed in our study (p=0.047) indicates an improvement in the temporal distribution of impact load and demonstrates that the protective mechanism highlighted by Bisciotti is reflected in real-world performance. This adaptation could provide critical protection, particularly during the “high RFD” moments (the first 50 ms of landing) when Anterior Cruciate Ligament (ACL) injuries are most observed (Faivre et al., 2025; Gaugg et al., 2025).

The balance of eccentric hamstring strength between the legs is also of vital importance in injury prevention. Benson et al. (2024) and Riego-Ruiz et al. (2026) found that hamstring strength asymmetry in soccer players disrupts landing mechanics, leading to “hard landing” strategies and, consequently, increased joint loads. In our study, the reduction of landing forces to a lower level following the training camp supports the hypothesis proposed by Benson et al. (2024) that risk factors associated with asymmetry can be eliminated through the training period. Additionally, the force balance between the dominant and non-dominant legs, as emphasized by Lutz et al. (2022), is considered the primary factor determining our athletes’ stabilization strategies during landing (Griffin et al., 2015; Nunes et al., 2018). The correlation results presented in **Table 6** reveal a significant positive relationship between jump height and peak landing force (r = 0.524) and RFD (r = 0.412). This finding indicates that every increase in jump performance among elite soccer players is accompanied by an increase in the mechanical stress and loading rate on the knee joint during the landing phase. The moderate positive correlation found between jump height and peak landing force (r = 0.524) underlines a critical dilemma for elite sports conditioning: higher-performing players who achieve superior vertical displacements are simultaneously exposed to heightened mechanical stress during deceleration. To offset this elevated injury risk, coaches must deliberately program progressive eccentric overloads and deceleration mechanics into seasonal routines. As highlighted by Islam et al. (2024), progressive eccentric paradigms like the Nordic hamstring curl act as a primary remedy for lowering structural injury rates by modifying muscle architecture and fascicle length. Furthermore, since dynamic jumping performance heavily relies on well-rounded physical capacities throughout a macrocycle (Mola et al., 2025), integrating specific landing absorption drills ensures that the metabolic and physical gains of a pre-season camp are safely translated into functional field performance without compromising joint integrity. Consequently, it is a scientific reality that athletes with improved performance require a much more advanced eccentric control capacity to manage these increased loads.

**Table 6:** Relationship between vertical jump height (CMJ) and landing phase parameters.

Parameters	Jump height (cm) - correlation (r)	p Value
Peak Landing Force (N)	0.524	0.003*
Landing RFD (N/s)	0.412	0.021*
Landing Impulse (Ns)	0.185	0.318

*indicates statistical significance. *Statistically significant correlation at the p<0.05 level.

Shock absorption occurring within the first 100 milliseconds of the landing phase is directly related to how quickly and coordinately the central nervous system activates the muscles (Studnicki et al., 2025). Hosseini et al. (2025) highlighted the ability of eccentric strength to transfer to functional tasks by demonstrating that Nordic Hamstring exercises provide significant improvements in complex motor tasks such as balance and agility. The increase in impulse observed in our findings indicates that athletes can distribute energy over a broader time frame thanks to the neuromuscular control they acquire following training (Greig & Siegler, 2009; Hosseini et al., 2025; Severo-Silveira et al., 2018). This aligns with the view expressed by Sinulingga and Zemková (2025) that the timing of peak force generation is the key to success in dynamic tasks.

The scientific validity of the measurement methods used in this study reinforces the reliability of our findings. Arp et al. (2024) and Connor et al. (2022) have validated the accuracy of the force platform and dynamometer systems used in capturing strength asymmetries and explosive power parameters in professional soccer players. Similarly, Lee et al. (2017) and Thotawatta et al. (2023) have demonstrated that force platform data serve as objective criteria for the long-term monitoring of athletes.

The fact that the relationship between eccentric hamstring strength and landing kinetics remains weak to moderate can be explained by the distinct neuromuscular nature of these two tasks. While the Nordic Hamstring Test is an isolated, low-speed task, landing after a vertical jump is a dynamic action occurring within milliseconds that requires the involvement of hip extensors. This suggests that the improvement in landing quality following the training camp may stem from the neuromuscular adaptation and motor learning effects induced by the combined pre-season camp program—which integrated progressive NHE protocols alongside team conditioning, tactical drills, and sport-specific plyometric activities—rather than an isolated absolute increase in hamstring strength alone.

## Conclusion

This study examined the effects of a 6-week preseason training camp on eccentric hamstring strength and landing biomechanics following a vertical jump in professional soccer players. The findings revealed that the training camp program resulted in both a reduction in body weight and a significant increase in normalized eccentric hamstring strength (p=0.021). This combination of weight loss and strength gain contributed to a significant reduction in peak loads during landing. However, the significant 6.5% reduction observed in normalized peak landing force (p=0.001) suggests that this improvement may stem not directly from an increase in muscle strength, but rather from neuromuscular coordination and motor learning adaptations gained during the training camp. In conclusion, pre-season camps should not focus solely on strength gains; neuromuscular training that optimizes landing mechanics to manage the mechanical loads associated with increased performance should be integrated into training programs. Future studies are recommended to examine the specific muscle activation timing and kinematic changes underlying this improvement. Another critical finding of the study is the positive correlation between vertical jump height and mechanical stress (Peak Force and RFD) during landing. This indicates that athletes with improved athletic performance (jump height) are exposed to higher impact loads during landing and require an enhanced neuromuscular control capacity to absorb these loads. On the other hand, the fact that the relationship between eccentric hamstring strength and landing parameters remains weak to moderate suggests that landing quality depends not only on the strength of an isolated muscle group but also on the coordinated energy absorption strategy of the hip, knee, and ankle complex.

In conclusion, preseason training camps should not focus solely on regional muscle hypertrophy or absolute strength gains. Instead, the functional benefits of a structured macrocycle should be viewed as a multifaceted adaptation encompassing concurrent changes in body composition, improved neuromuscular coordination, and acute motor learning. Neuromuscular training models that optimize multi-joint landing mechanics to safely absorb the increased physical stress associated with athletic performance should remain fundamental components of elite athletes’ training programs. Future studies are recommended to investigate the effects of hamstring strength, as well as the timing of activation and the (Hamstring: Quadriceps) ratio of hip extensors, on landing kinetics.

### Limitations of the study and future research

Certain methodological limitations of this study should be carefully considered. First, the study design does not include a true randomized control group; this limits our ability to establish direct causal relationships. Therefore, the observed improvements should be interpreted not as definitive causal effects resulting from the training intervention, but rather as an association. Second, although a progressive Nordic Hamstring Exercise (NHE) protocol was implemented, the total external and internal training volumes (such as tactical periodization, daily GPS loads, and sport-specific plyometric exposure) during the professional preseason camp were not precisely distinguished or measured. Third, our biomechanical assessment of the landing phase of the jump was based entirely on force (kinematic analysis via force plates). Since kinematic data (such as specific hip, knee, and ankle joint angles or dynamic valgus) were not included, we cannot fully explain the precise mechanical causes of the recorded kinetic changes (Alanazi et al., 2021).

Furthermore, the lack of electromyographic (EMG) data limits our ability to analyse muscle activation timing, pre-activation of muscles, or simultaneous contraction strategies. The landing following a vertical jump is a multi-joint, dynamic movement that occurs within milliseconds and requires the active and coordinated participation of the hip extensors (Gluteus Maximus) and the quadriceps group alongside the hamstring muscles. In this process, the role of the hamstrings may be limited to adjusting the momentary stability (stiffness) of the knee joint rather than providing absolute braking. Consequently, landing quality depends on the coordinated energy absorption strategy of the entire lower extremity kinetic chain rather than an isolated muscle group. To cross-validate these kinetic adaptations and better elucidate how changes in hamstring strength reflect changes in joint angles and dynamic joint stiffness, future studies utilising a randomised controlled design, 3D motion analysis systems (e.g., Vicon), electromyography, and allometric scaling models are strongly recommended (Sheldon et al., 2025; Studnicki et al., 2025).

## Data Availability

The original contributions presented in the study are included in the article/supplementary material. Further inquiries can be directed to the corresponding author.
